# Pneumonia in Patients with Chronic Kidney Disease Admitted to Nephrology Department of a Tertiary Care Center: A Descriptive Cross-sectional Study

**DOI:** 10.31729/jnma.7074

**Published:** 2021-10-31

**Authors:** Abhishek Pant, Astha Prasai, Anmol Kumar Rauniyar, Laxman Adhikary, Krity Basnet, Tunam Khadka

**Affiliations:** 1 Department of Medicine, Grande International Hospital, Dhapasi, Kathmandu, Nepal; 2 Department of Medicine, Hospital for Advanced Medicine and Surgery Hospital, Dhumbarahi, Kathmandu, Nepal; 3 Kathmandu Medical College, Sinamangal, Kathmandu, Nepal; 4 Department of Nephrology, Kathmandu Medical College, Sinamangal, Kathmandu, Nepal

**Keywords:** *ckd*, *pneumonia*, *hemodialysis*

## Abstract

**Introduction:**

Chronic Kidney Disease is an independent risk factor for pneumonia. The risk of hospitalization, Intensive Care Unit and ventilator requirement, in-hospital death is high in pneumonia patients with chronic kidney disease. This study aims to find the prevalence of pneumonia in patients with chronic kidney disease admitted to nephrology department of a tertiary care center.

**Methods:**

A descriptive cross-sectional study was conducted among all the hospital records of pneumonia patients with Chronic Kidney Disease admitted to the Nephrology department between April 2019 and April 2021. Ethical clearance was obtained from the Institutional Review Committee of same institute (Reference number: 0505202106). Statistical Package for the Social Sciences version 20 was used for analysis. Point estimate at 95% Confidence Interval was calculated along with frequency and proportion for binary data.

**Results:**

Of the total 407 patients with Chronic Kidney Disease, 78 (19.1%) (95% Confidence Interval= 15.28-22.92) had pneumonia. Among the 78 pneumonia patients, 17 (21.8%) were Stage 3, 13 (16.7%) Stage 4 and 48 (61.5%) Stage 5 of chronic kidney disease. Forty Seven (60.3%) required Intensive Care Unit (ICU), 19 (24.4%) required ventilator and 22 (28.2%) of the patient expired in hospital. The most commonly isolated organisms were Severe Acute Respiratory Syndrome Coronavirus 2 which was 13 (16.6%) followed by Strepotococcus pneumoniae which was 8 (10.2%).

**Conclusions:**

The prevalence of pneumonia in Chronic Kidney Disease was observed higher in our study compared to other studies.

## INTRODUCTION

Infections are a major cause of morbidity and mortality in Chronic Kidney Disease (CKD) patients. The relationship is mutual: not only infections are severe and difficult to manage in CKD, but infections also contribute to the progression of CKD and complicate its management.^1^ Lower respiratory tract infections e.g. Pneumonia are common occurrences in CKD patients and are associated with increased risk of hospitalization, cardiovascular events and mortality.^2^

CKD has long been considered an independent risk factor for pneumonia. The risk of pneumonia is up to 1.97 fold higher in CKD patients-1.4 times higher for outpatient pneumonia and even higher, i.e., 2.17 times for inpatient pneumonia compared with patients without CKD.^2^

This study aims to find the prevalence of pneumonia in patients with chronic kidney disease admitted to nephrology department of a tertiary care center.

## METHODS

In this descriptive cross-sectional study, hospital records of the patients with Chronic Kidney Disease who were admitted under the Nephrology department of Kathmandu Medical College and Teaching Hospital (KMCTH) from April 2019 to April 2021 were accessed. Ethical approval was taken from the Institutional Review Committee of KMCTH before data collection (Reference number: 0505202106). All patients more than 18 years of age, CKD stage 3 and above with evidence of pneumonia were eligible for the study. All the samples within the last 2 years were collected and the sample size was calculated using the given formula:

Sample size (n) = Z^2^ × p × q / e^2^

  = (1.96)^2^ × 0.103 × 0.897 / (0.03)^2^

  = 394.37

  = 395

where,

Z = 1.96 at 95% confidence interval
p = prevalence of pneumonia with CKD was found to be 10.3%.^3^
q = 1-p
e = margin of error, 3%

However a total of 407 hospital records of patients with CKD were evaluated. The Cockcroft-Gault equation for creatinine clearance was used for calculating estimated Glomerular Filtration Rate (eGFR). Stages of CKD were established using the Kidney Disease Improving Global Outcomes (KDIGO) criteria. Collected data was entered in Microsoft Excel and Statistical Package for Social Sciences version 20 was used for data analysis. Point estimate at 95% confidence interval and descriptive statistics were calculated.

## RESULTS

Out of the total 407 patients with CKD admitted to the Nephrology department, 78 (19.1%) (95% Confidence Interval= 15.28-22.92) had pneumonia. Among the patients with pneumonia, 51 (65.4%) were males and 27 (34.6%) were females. Pneumonia was further categorized based on the CKD stages - 17 (21.8%) cases of pneumonia were reported in Stage 3 CKD, 13 (16.7%) in Stage 4 and 48 (61.5%) in Stage 5. The mean duration of CKD in these patients was 22.99±24.28 months (minimum 3 months, maximum 9 years). Forty five (57.7%) of the CKD patients were under maintenance hemodialysis at the time of admission. The mean length of hospital stay was 8.97±9.47 days (minimum 1 day, maximum 50 days). 47 (60.3%) of the cases required ICU and 19 (24.4%) required ventilation for the treatment of pneumonia. Death was reported in 22 (28.2%) cases (Table 1).

**Table 1:** Frequency by ^*^CKD grades.

CKD Stage	^†^ICU n (%)	Ventilator n (%)	Death n (%)
Stage 3	8 (17.0)	5 (26.3)	5 (22.7)
Stage 4	9 (19.1)	2 (10.5)	2 (9.0)
Stage 5	30 (63.8)	12 (63.1)	15 (68.1)
Total	47 (100)	19 (100)	22 (100)

* CKD: chronic kidney disease,

† ICU: intensive care unit.

The mean age of the cases was 60.43±17.55 years (minimum 20 years, maximum 92 years). Forty-one (52.6%) of the cases were diabetic and 28 (35.9%) were active smokers.

Severe Acute Respiratory Syndrome Coronavirus 2 (SARS-CoV-2) was the most commonly isolated organism followed by Streptococcus pneumoniae and Klebsiella pneumoniae (Table 2).

**Table 2:** Microscopic examination of sputum sample (n = 78).

Organism	n (%)
^*^SARS-CoV-2	13 (16.6)
Streptococcus pneumoniae	8 (10.2)
Klebsiella pneumoniae	7 (8.9)
Acinetobacter baumannii	3 (3.8)
Pseudomonas aeruginosa	2 (2.5)
Escherichia coli	1 (1.2)
Coagulase negative Staphylococcus	1 (1.2)
No organism isolated	13 (16.7)
Not tested	30 (38.5)

* SARS-CoV-2: Severe Acute Respiratory Syndrome Coronavirus 2

A combination of Beta-Lactam and Macrolide was the most commonly used antibiotic regimen for the treatment of pneumonia in CKD under maintenance hemodialysis and not under dialysis as described (Table 3).

**Table 3:** Antibiotic use based on ^*^CKD management

Antibiotics	^†^NUD n (%)	^‡^MHD n (%)
Monotherapy	5 (15.6)	4 (8.7)
B-Lactam + Macrolide	12 (37.5)	13 (28.3)
B-Lactam + Fluoroquinolone	2 (6.3)	5 (10.9)
B-Lactam + Anti-pseudomonal	1 (3.1)	3 (6.5)
B-Lactam + Clindamycin	3 (9.4)	5 (10.9)
Multidrug Therapy	9 (28.1)	16 (34.8)
Total Patients	32 (100)	46 (100)

* CKD: chronic kidney disease

† NUD: not under dialysis

‡ MHD: maintenance hemodialysis

## DISCUSSION

CKD compromises the host's ability to fight, especially affecting the cell-mediated immune system. The role of uremia in increasing the susceptibility of CKD patients to infections has also been well studied by researchers. Uremia has been linked to impairment in neutrophil^4,5^ and lymphocyte functioning.^6,7^ Patients who receive hemodialysis for the treatment are at further increased risk of infection as it requires vascular access, exposure to injectable medications, dialyzers, and equipment among others.^8^’^9^

Studies suggest that the pneumonia-related mortality rate in CKD could be as high as 14-16 times as compared to the general population.^2^ The patient receiving hemodialysis are twice as susceptible to pneumonia compared to other forms of renal replacement therapy (RRT) such as renal transplant and peritoneal dialysis ^10^ and the mortality rate is 14-16 times higher than the general population.^11^

The prevalence of pneumonia in our study was significantly higher than another similar national study.^3^ One possible reason behind this could be the inclusion of Covid-19 pneumonia in our study that was prevalent during the duration of our study. The mean age of the study was 60.43±17.55 years which was quite similar to a study conducted in Taiwan with a mean age of 64±16.0.^2^ Forty one (52.6%) of the patients were diabetic similar to the report from USRDS.^12^ Our study reports a high frequency of ICU requirement 47 (60.3%) and ventilator requirement 19 (24.4%) in CKD patients with pneumonia. This was significantly higher than the study by Viasus, et al. who reported ICU requirement of 8.9% and ventilator requirement of 7.9%.^13^ The average length of hospital stay for pneumonia in a study in Spain was 5-7 days ^14^ and 5.4 days according to US-Healthcare and Cost Utilization Project which is lower than the mean of 8.97±9.47 days in our study. Also, the in-hospital mortality was higher at 28.2.% in our study as compared to the report by James et al. which reported 21% death within 30 days of hospitalization with pneumonia.^15^ This is probably due to the increased severity of pneumonia in CKD patients although the difference in the quality of care between the developed and developing nations might be a factor here as well. The same study also reported that with a decrease in eGFR from 60 to less than 30/ml/min/1.73m^2^, the incidence rate of death after hospitalization with pneumonia increased from 0.17 to 8.55 deaths/1000.^15^ This was consistent with our study, where the highest death was reported among CKD Stage 5-15 (68.1%). SARS-CoV-2 was the most commonly isolated organism with a total of 13 (16.6%). Covid-19 pneumonia is a newly emerging disease with very limited data. The most recent studies have suggested an increased severity of COVID-19 disease, length of hospitalization, ICU admission and mortality observed in CKD patients.^16^ We observed 5 deaths (38.4%), 8 ICU admissions (61.5%), 3 ventilator requirements (23.0%) in this subgroup. Ten out of the thirteen SARS-CoV-2 pneumonia were reported in CKD Stage 5. A study by Carlson, et al. reported a similar pattern where they reported that eGFR was inversely associated with the rate of hospital diagnosed COVID-19.^17^ However, the sample size is too small for any conclusions and the authors advocate for a separate study on this emerging topic. Otherwise, the most commonly isolated organism was Streptococcus pneumoniae similar to other studies^8^ with a frequency of 8 (10.2%).

We also tried to assess the pattern of antibiotics use in hospitalized pneumonia patients with CKD receiving maintenance hemodialysis and compared it to the CKD patients not under dialysis. Two-drug combination therapy was mostly preferred in both maintenance hemodialysis and not under dialysis groups which were 26 (56.5%) and 18 (56.2%) respectively. The most commonly used dual therapy in either group was a combination of Beta-Lactam and Macrolide which was 13 (28.3%) and 12 (37.5%) in maintenance hemodialysis and not under dialysis groups respectively. The use of multidrug (more than 2 antibiotics) for the treatment of pneumonia was higher in CKD patients under maintenance hemodialysis which was 16 (34.8%) than pneumonia patients with CKD and not under dialysis which was 9 (28.1%). This is probably because hemodialysis is associated with increased infection-related comorbidities as well as multidrug-resistant organisms.^8,11^

This study has a few limitations. First, this is a retrospective study conducted in a single institution and many data were subjectively evaluated. Secondly, the severity index of pneumonia (PSI) was not taken into account due to the lack of medical records. This could affect certain outcomes especially the length of hospital stay, ICU and ventilator requirement and in-hospital mortality. Finally, not all the medical records had culture reports, therefore the isolated organisms reported in our study may not represent the true picture. Therefore careful interpretation and application of the findings of this study are advised.

## CONCLUSIONS

In conclusion, the prevalence of pneumonia in CKD was much higher in our study compared to another similar national study.
